# Yeast Models and Molecular Mechanisms of Neurodegenerative Diseases 2.0

**DOI:** 10.3390/ijms232415821

**Published:** 2022-12-13

**Authors:** Joanna Kaminska, Teresa Zoladek

**Affiliations:** Institute of Biochemistry and Biophysics PAS, 02-106 Warsaw, Poland

One of the goals of human genetics is to discover the variants that contribute to human diseases. With the rapid development and widespread use of next-generation sequencing (NGS), huge datasets of genomic sequences have been collected. However, there is often a lack of knowledge regarding the relationship between sequence variants and phenotypes, based on clinical observations and experimental evidence. The number of variants obtained from the NGS of trios (patients and parents) is more than 50,000 and can be reduced to just a dozen variants of potential interest, but selecting the best candidate among these variants requires the use of different models as well as in vivo, in vitro, and in silico studies [1].

This Special Issue of the International Journal of Molecular Sciences (MDPI), entitled “Yeast Models and Molecular Mechanisms of Neurodegenerative Diseases 2.0,” presents research on the pathogenicity of gene variants in a subset of rare neurological diseases. Studies are presented on the mutations in *IGHMBP2*, which cause spinal muscular atrophy with respiratory distress type 1 (SMARD1) [2], and in the four *VPS13* genes (*VPS13A–D*) that cause in chorea-acanthocytosis (recently renamed VPS13A disease [3], Cohen syndrome, early-onset Parkinson’s disease, and spastic ataxia/paraplegia, respectively [4]. Additionally, some studies focus on the identification of Vps13 protein localization determinants, and a review summarizes the studies showing new phenotypes of the *vps13*∆ mutant and research that aims to identify potential therapies [5]. Moreover, a review on α-arrestins is included in this collection. These are adaptor proteins that link protein substrates with ubiquitin ligases, modifying them with ubiquitin chains for direct degradation [6]. Mutations in genes encoding α-arrestins are connected to several human disorders, including neurological diseases.

SMARD1 is an example of a disease that requires a timely diagnosis because its symptoms are severe, such as respiratory failure appearing early in life and progressive muscle weakness and wasting. This rare, heritable neurodegenerative disease is caused by mutations in the highly polymorphic *IGHMBP2* gene, with many variants that have an unknown effect on protein function [7]. The *IGHMBP2* gene encodes immunoglobulin mu-binding protein 2 (IGHMBP2), which is a helicase of unknown cellular function. Rzepnikowska et al. [2] generated humanized yeast cells, a model of SMARD1, to predict *IGHMBP2* variants’ pathogenicity. Through this approach, the authors demonstrated that the cDNA of the human *IGHMBP2* gene can functionally replace the homologous *HCS1* gene, which allows the impact of different mutations on IGHMBP2 protein functionality to be assessed. The authors demonstrated also a correlation between the phenotype estimated from in vitro studies described in the literature and results from a yeast model, which indicate that such humanized yeast could be used as a quick and simple method to distinguish between pathogenic and nonpathogenic mutations identified in the *IGHMBP2* gene.

Over the past few years, there has been increasing interest in the proteins of the Vps13 family, especially since they are linked to neurodegeneration. In 2018, Prof. de Camilli and collaborators succeeded in showing, in vitro, that the N-terminal fragment of the yeast Vps13 protein transports bulk lipids between liposomes [8]. Subsequent studies have discovered more proteins that have a similar structure and behavior, e.g., transferring lipids [9]. However, it is unknown how this defect in lipid transfer is linked to the pathogenesis of different diseases. The yeast model, with a single *VPS13* gene encoding a protein highly similar to the human VPS13A–D proteins, seems to be an excellent model to study the impact of non-null alleles caused by missense mutations on the Vps13 protein function in different processes and, more generally, the role of Vps13 proteins and their regulation. This was the concept behind the results presented by Park et al. [4] where mutations were introduced into the yeast *VPS13* gene to cause amino acid substitutions at cognate positions to those of VPS13A, B, C, and D, associated with human diseases, with the aim of identifying critical residues for Vps13 function [4]. Phenotypic analyses showed the genetically separable functions of the Vps13 protein in processes such as the vacuolar sorting of carboxypeptidase Y (CPY), sporulation efficiency, and the promotion of ER-phagy. In this study, the authors also provided evidence that adaptors at the endosome mediate the activity of VPS13 in vacuolar sorting, while in another study by Kołakowski et al. [10], the authors documented that the localization of the yeast Vps13 protein to the Golgi membranes is important for CPY sorting. These studies also show that Golgi localization is caused by the interaction of the Vps13 C-terminal plekstrin homology (PH)-like domain with the Arf1 GTPase protein. Moreover, research shows that the PH-like domain interacts with a phosphoinositol 4,5-bisphosphate lipid. The similar properties of the PH-like domain of homologous VPS13A were demonstrated, suggesting that PH-like domains are determinants of the VPS13 proteins’ localization to the Golgi apparatus in both yeast and humans. These publications are accompanied by a review in which the authors summarize recent studies aiming to determine the new phenotypes associated with the yeast *vps13*Δ and *vps13-I2749* mutations and find potential new drug targets to treat patients. These studies describe the results of yeast screens for multicopy and chemical suppressors, which point to areas for further research using other higher eukaryotic models of diseases associated with the absence of functional individual VPS13 proteins, as well as new therapeutic targets, such as calcium signaling and copper and iron homeostasis. Moreover, the identified pharmaceuticals have the potential to be repurposed for these diseases [5].

The last review presents a protein family of α-arrestins that are conserved from yeast to humans; their predominant function is the selective identification of membrane proteins, such as receptors and transporters, for ubiquitination via interacting with the ubiquitin ligases of the Rsp5/NEDD4 family and directing them for vacuolar/lysosomal degradation. This is an important process for maintaining membrane protein homeostasis as well as global cellular metabolisms, including lipid metabolism. α-Arrestins are highly regulated by phosphorylation and other modifications in response to different stimuli, such as stress and the availability of nutrients, as was mainly determined by observations from the yeast model. However, several functions of α-arrestins in animal models were recently characterized, including redox homeostasis regulation, innate immune response regulation, etc. α-Arrestins were also implicated in health disorders, including neurodegenerative diseases, pointing to the importance of proper protein degradation in neuronal cell physiology [6].

This Special Issue complements its predecessor with a similar title and presents advances in research on VPS13 proteins and VPS13-related diseases. It also introduces two new topics. We hope that the presented findings will help the scientific community discover new research directions, such as investigating how IGHMBP2 and VPS13 proteins are degraded and whether their impaired degradation may contribute to the pathogenesis of particular diseases. This subject, as well as crosstalk between lipid transport and protein degradation, remains an exciting topic for further research aiming to better understand the molecular pathogenesis of neurodegenerative disorders, which may allow for the development of new treatments for patients who are waiting for them.
